# The Relationship between Running Power and Running Economy in Well-Trained Distance Runners

**DOI:** 10.3390/sports6040142

**Published:** 2018-11-06

**Authors:** Casey L. Austin, James F. Hokanson, Peter M. McGinnis, Steve Patrick

**Affiliations:** 1 Proehl Exercise Physiology Laboratory, Kinesiology Department, State University of New York at Cortland, Cortland, NY 13045, USA; casey.austin@cortland.edu (C.L.A.); peter.mcginnis@cortland.edu (P.M.M.); 2 Athletics Department, State University of New York at Cortland, Cortland, NY 13045, USA; steve.patrick@cortland.edu

**Keywords:** power, running, running economy, running power meter, Stryd, distance runners, wearables, accelerometry

## Abstract

A novel running wearable called the Stryd Summit footpod fastens to a runner’s shoe and estimates running power. The footpod separates power output into two components, Stryd power and form power. The purpose of this study was to measure the correlations between running economy and power and form power at lactate threshold pace. Seventeen well-trained distance runners, 9 male and 8 female, completed a running protocol. Participants ran two four-minute trials: one with a self-selected cadence, and one with a target cadence lowered by 10%. The mean running economy expressed in terms of oxygen cost at self-selected cadence was 201.6 ± 12.8 mL·kg^−1^·km^−1^, and at lowered cadence was 204.5 ± 11.5 mL·kg^−1^·km^−1^. Ventilation rate and rating of perceived exertion (RPE) were not significantly different between cadence conditions with one-tailed paired t-test analysis (ventilation, *p *= 0.77, RPE, *p *= 0.07). Respiratory exchange ratio and caloric unit cost were significantly greater with lower cadence condition (respiratory exchange ratio, *p *= 0.03, caloric unit cost, *p *= 0.03). Mean power at self-selected cadence was 4.4 ± 0.5 W·kg^−1^, and at lowered cadence was 4.4 ± 0.5 W·kg^−1^. Mean form power at self-selected cadence was 1.1 ± 0.1 W·kg^−1^, and at lowered cadence was 1.1 ± 0.1 W·kg^−1^. There were positive, linear correlations between running economy and power (self-selected cadence and lower cadence, *r *= 0.6; the 90% confidence interval was 0.2 to 0.8); running economy and form power (self-selected cadence and lower cadence *r *= 0.5; the 90% confidence interval was 0.1 to 0.8). The findings suggest running economy is positively correlated with Stryd’s power and form power measures yet the footpod may not be sufficiently accurate to estimate differences in the running economy of competitive runners.

## 1. Introduction

Use of wearable technologies (wearables) like Global Positioning System (GPS) watches, fitness trackers, and pedometers has increased in recent years [1]. Wearables have been cited as an advancement in fitness tracking since they provide more precise data than classic self-assessment can [2]. Wearable use in performance settings has increased as well, with sports teams and high-level athletes taking advantage of technological advancements to track data over time to pinpoint areas for improvement [3]. Recent advancements in wearables have introduced novel metrics that may be useful to athletes trying to improve their performance. One of these metrics is power.

Cycling power meters have been available for years. Cyclists use these in training and racing and the relationships between cycling power output and oxygen consumption (VO_2_) [4], cycling economy [5], and cycling efficiency [6] have been established. Most cycling power meters measure power from the force or torque exerted by the cyclist on the pedals or crank and the angular velocity of the crank. Not all of an athlete’s metabolic power (VO_2_) is transmitted into useful mechanical power (cycling power) [7]. Most of the metabolic power or oxygen cost is used to produce heat, to maintain resting metabolic rate, to produce negative work via eccentric muscular contractions, and to produce unnecessary movements if the athlete has poor technique [7]. 

A variety of methods for measuring or estimating running power have been explored. In these methods different assumptions were made when calculating power and the power calculations required ground reaction force data or kinematic data for input. Due to these differences in assumptions and input data, the values resulting from the power calculations varied widely [8,9,10,11,12]. A well-accepted definition of running power does not exist [11]. However, a few recent wearables for runners include estimates of running power. The Stryd Summit (Stryd, Boulder, CO, USA) is one of these. The Stryd Footpod is composed of a triaxial accelerometer, a gyroscope, and a barometer embedded into a small shoe-mounted chip. The Footpod estimates running power as well as measures running pace, distance, vertical oscillation, cadence, leg spring stiffness and ground contact time. The Footpod separates running power output into two components: power and form power. Power appears to represent the power output related to changes in the athlete’s horizontal motion, whereas form power appears to represent the power output related to changes in the athlete’s vertical motion. The Footpod uses undisclosed algorithms to calculate power and form power from the kinematic data it collects from the movements of the user’s foot.

Running economy is defined as the energy demand required to sustain a submaximal velocity [13]. Running economy in terms of cost of transport (ml O_2_·kg^−1^·km^−1^) is calculated using measurements of steady-state oxygen consumption, participant body mass, and distance traveled [14,15]. Running economy may also be calculated in terms of caloric unit cost using respiratory exchange ratios [14,16]. 

Running economy is considered an essential component of distance running performance and is modulated by both biomechanical and physiological factors [17,18]. It is generally considered a better predictor of performance than VO_2max_ among similarly capable distance runners [17]. Since running economy is related to oxygen consumption at submaximal velocities [16], improving running economy means lowering oxygen consumption at a given velocity, which may lead to improved performance [13,19,20,21]. Therefore, it is desirable for competitive distance runners to improve their economy by lowering the energy cost of running.

Running economy is related to biomechanical factors including vertical oscillation, ground contact time, stride length, and stride frequency [22,23]. It has been shown that manipulation of stride frequency or stride length can lead to a change in running economy [24,25,26]. Most notably, De Ruiter et al. [25] measured a significant difference in running economy between self-selected cadence and altered cadences at ± 6%, ± 12%, and ± 18%.

Measurement of running economy requires expensive equipment (metabolic analyzer) that most runners do not have free access to. As wearable devices have become more sophisticated, athletes and coaches have had access to more data [3]. Wearables present runners with data from daily training that is typically collected in a lab (e.g., vertical oscillation, ground contact time, cadence). While wearable technology use has increased, there have been few studies examining the use of new proprietary measures as an alternative to lab-collected data. Aubry, Power, and Burr [27] assessed power measured by a Stryd Pioneer chest strap as a surrogate for metabolic cost (VO_2_). The researchers found that power and VO_2_ had a weak positive relationship (*r *= 0.29). Since running economy is considered central to running performance [17], an alternative metric (power and form power) measured by a wearable sensor (footpod) could be useful to competitive runners. 

It is unknown if power as measured by the Footpod has a stronger relationship with running economy than power as measured by the Stryd Pioneer chest strap. Furthermore, it is unknown if changes in cadence will cause significant change in power or form power, as they can with running economy [24,25,26]. It was hypothesized that with a decrease in cadence from a self-selected value, power and form power would increase significantly along with running economy. Furthermore, it was hypothesized that power and form power would have a positive relationship with running economy. It was predicted that physiological measures of ventilation, respiratory exchange ratio, and psychological measure of rating of perceived exercitation (RPE) would be significantly greater with a lower running cadence at the same treadmill speed. The purpose of this study was to measure the correlation between the Footpod power metrics (power, form power) and running economy in well-trained collegiate distance runners at self-selected and lowered cadences.

## 2. Materials and Methods

### 2.1. Participants

A total of 17 participants were recruited and took part in the study (nine males, eight females). Participants were either current or alumni members of the university’s cross country or track and field team. The study was reviewed and approved by the university’s Institutional Review Board. Informed consent was obtained, then running history and physical activity readiness questionnaires were completed before the running trials. 

The focus of the investigation was on testing the efficacy of the Footpod power metrics as measures related to running economy. Participant VO_2max _values were estimated using recent (within 12 months) race performance times (distances from 800 m to marathon) [28,29]. Estimated VO_2max_ values were then utilized to prescribe a workload estimated to be at lactate threshold pace corresponding to 85–89% of velocity at VO_2max _(vVO_2max_), which was a training intensity all subjects were familiar with [28,29]. vVO_2max_ has been established as a measure that more closely correlates with running performance than either running economy or VO_2max_ individually [30,31]. All participants self-reported running at least 25 miles per week. Participants were excluded if a minimum estimated VO_2max_ value that correlated with an estimated VO_2max_ was not exceeded (44 mL·kg^−1^·min^−1^ for women; 50 mL·kg^−1^·min^−1 ^for men). Participant characteristics are reported in Table 1.

### 2.2. Instruments

A Parvo Medics TrueOne 2400 metabolic analyzer (Parvo Medics, Sandy, UT, USA) was used to record gas exchange measures. Gas exchange data were recorded on a Dell desktop computer. Prior to each testing session, the metabolic analyzer was powered on to warm up and then O_2_ and CO_2_ sensors were calibrated based on known gas tank concentrations and room air measurements. Flowmeter calibrations were then completed using a 3.0 L syringe. Stryd metrics were recorded by a Stryd Summit footpod (Stryd, Boulder, CO, USA) paired to a Garmin Fenix 3 watch (Garmin Ltd., Olathe, KS, USA) with the Stryd Run Data Field. Testing was completed on a Trackmaster TMX425c treadmill (Trackmaster Treadmills, Newton, KS, USA).

### 2.3. Procedures

Prior to testing, participants were given a brief overview of the protocol. Participants then put on the metabolic analyzer mouthpiece and nose clip. The Footpod was attached to the runner’s left shoelace equidistant from the participant’s malleolus and the toe of their shoe. Participant height and weight values were entered in the Stryd Android app and Garmin watch.

Participants completed a two-stage discontinuous running protocol. Data collection began during a five-minute warm-up at a self-selected “easy” pace. Participant self-selected cadence was then determined during a one-minute run at the participant’s assigned threshold pace immediately after the five-minute warm-up. Following the warm-up and self-selected cadence measurement, participants took a three-minute standing rest on the treadmill. During the rest, researchers calculated a cadence value 10% lower than the participants’ self-selected cadence for them to replicate during the experimental protocol. A coin was flipped to determine the order of testing protocol stages. 

For the self-selected cadence stage, participants ran four minutes at assigned threshold pace while attempting to match their cadence with a metronome chirping at their self-selected cadence from the warm-up period. For the lower cadence stage, participants ran four minutes at assigned threshold pace while attempting to match their cadence with the metronome set to a value 10% lower than their self-selected cadence (actual measured cadence was ~4% lower, see results section).

After the rest period, participants began the first stage of the experimental protocol (self-selected or lowered cadence). Upon completion of their first stage, participants rested for three minutes. After the first stage rest period, participants began their second stage (self-selected or lowered cadence). Upon completion of the second stage, participants rested for one minute and data collection ceased. Ratings of perceived exertion (RPE) were recorded at the completion of the self-selected and lowered cadence running stages. Running economy (in terms of O_2_ cost and caloric unit cost), caloric unit cost, VO_2_, respiratory exchange ratio, ventilation, average power, average form power, and average cadence were calculated from the last minute of each running stage.

### 2.4. Statistical Analysis 

Statistical analysis was completed using IBM SPSS Statistics Version 23 (IBM Corp, Armonk, NY, USA). For all statistical analyses, significance was set at *p *< 0.05. One-tailed paired t-tests were run to determine if statistically significant differences occurred in variables of interest between the two cadence conditions. One-tailed bivariate correlation analyses were run on both cadence conditions separately to determine if there was a relationship between running economy, power, and form power. The Pearson correlation coefficient effect size was evaluated using Cohen’s scale [32]: <0.10, trivial; 0.10–0.29, small; 0.30–0.49, moderate; ≥0.50, large. Correlation coefficient confidence intervals (90% confidence level) were calculated using Fisher’s *z* transformation [33].

## 3. Results

Mean data (± standard deviation (SD)) for cadence, VO_2_, respiratory exchange ratio, RPE and ventilation separated by cadence condition are reported in Table 2. One-tailed paired t-tests were run on these variables to find if significant differences occurred between the two cadence conditions (self-selected or lowered cadence). The experimental design had the lowered cadence value significantly lower compared to self-selected, (*t*(15) = 6.573, *p *< 0.001). Physiological variables of VO_2_, *t*(16) = −1.856, *p *= 0.041, and respiratory exchange ratio, *t*(16) = −1.962, *p *= 0.034 were both significantly greater at the lowered cadence condition. There were no statistically significant differences between cadence conditions for the following variables: RPE, *t*(16) = −1.515, *p *= 0.075, and ventilation, *t*(16) = −1.495, *p *= 0.77.

Mean data (± SD) for running economy, caloric unit cost, power, and form power are reported in Table 3. One-tailed paired t-tests were conducted to determine if significant differences occurred between the two cadence conditions (self-selected or lowered cadence). The lowered cadence condition resulted in statistically significant greater running economy (*t*(16) = −2.017, *p *= 0.031), caloric unit cost (*t*(16) = −2.093, *p *= 0.027), power (*t*(16) = −6.349, *p* < 0.001), and form power (*t*(16) = −5.664, *p* < 0.001).

The results of the bivariate correlations of cadence, running economy, caloric unit cost, power, and form power are shown in Table 4 (self-selected) and 5 (lowered cadence). The correlations were run separately on data collected between the two cadence conditions. It should be noted that the target lowered cadence of 10% below self-selected cadence was not realized (self-selected = 179.6 ± 8.4; lowered cadence = 172.5 ± 9.5 strides·min^−1^); however, the decrease in cadence was significantly less during the lowered cadence condition (Table 3). 

Table 4 shows the relationships between cadence, running economy, caloric unit cost, and the Footpod’s power metrics (power and form power) for the self-selected cadence condition. Correlation coefficient values were statistically significant and negative for the following relationships: cadence and caloric unit cost (*r* = −0.7 to 0.0); cadence and form power (*r* = −0.9 to −0.6). Values were statistically significant and positive for the following relationships: running economy and caloric unit cost (*r* = 0.9 to 1.0); running economy and power (*r* = 0.2 to 0.8); running economy and form power (*r* = 0.1 to 0.8); caloric unit cost and power (*r* = 0.2 to 0.8); caloric unit cost and form power (*r* = 0.1 to 0.8); power and form power (*r* = 0.5 to 0.9).

**Figure 1:**
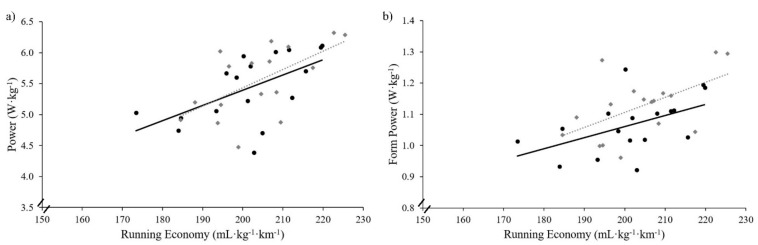

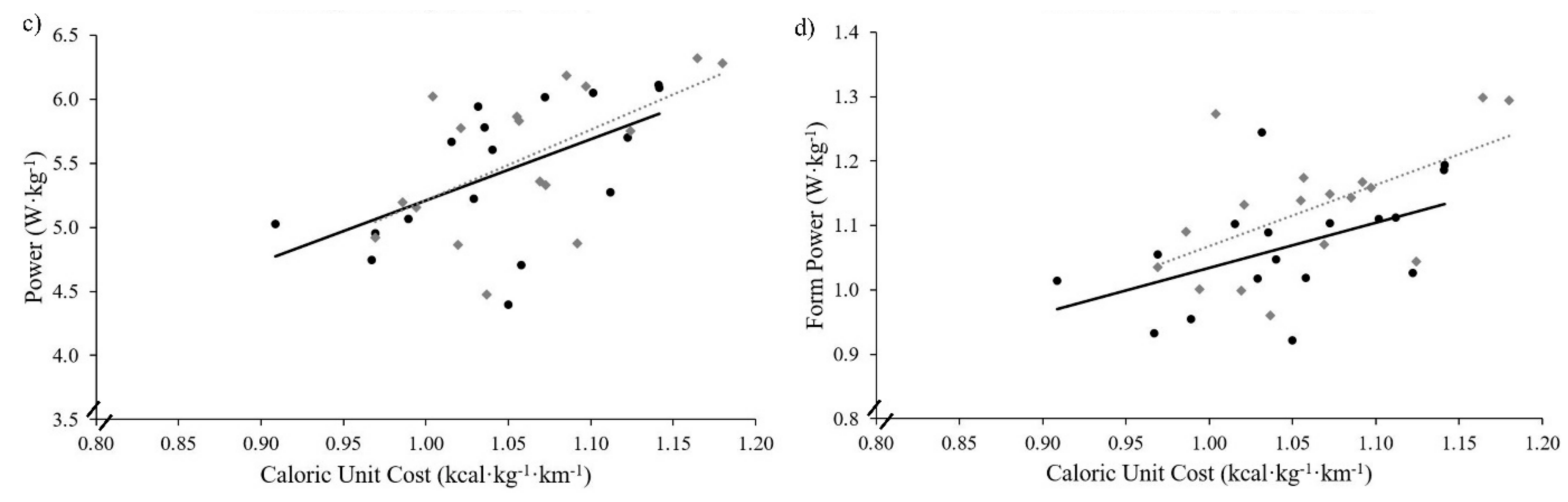
Correlation scatterplots for power metrics and running economy. Black circles and solid line of best fit represent self-selected cadence condition. Grey diamonds and dotted line of best fit represent lowered cadence condition. (**a**) Self-selected cadence power and running economy (*r* = 0.6); lowered cadence power and running economy (*r* = 0.6). (**b**) Self-selected cadence form power and running economy (*r* = 0.5); lowered cadence form power and running economy (*r* = 0.5). (**c**) Self-selected cadence power and caloric unit cost (*r* = 0.6); lowered cadence power and caloric unit cost (*r* = 0.5). (**d**) Self-selected cadence form power and caloric unit cost (*r* = 0.5); lowered cadence form power and caloric unit cost (*r* = 0.6).

In general, power was positively related to running economy for both cadence conditions. Correlation scatterplots for running economy and power metrics are shown in Figure 1. Table 5 shows the relationships between cadence, running economy, caloric unit cost, and the Footpod’s power metrics (power and form power) for the lowered cadence condition. Values were statistically significant and positive for the following relationships: running economy and caloric unit cost (*r* = 0.9 to 1.0); running economy and power (*r* = 0.2 to 0.8); running economy and form power (*r* = 0.2 to 0.8); caloric unit cost and power (*r* = 0.2 to 0.8); caloric unit cost and form power (*r* = 0.2 to 0.8); power and form power (*r* = 0.4 to 0.9). Correlation coefficient values (90% confidence limits) were statistically significant and negative for the following relationships: cadence and running economy (*r* = −0.8 to −0.1); cadence and caloric unit cost (*r* = −0.8 to −0.2); cadence and form power (*r* = −1.0 to −0.7). 

## 4. Discussion

The Footpod represents a novel method of estimating running power. The purpose of this study was to determine the relationship between established measures of running economy (oxygen cost, caloric unit cost) and Stryd’s proprietary power metrics (power, form power). Positive correlations (ranging from small to large) were measured between running economy and Stryd’s power and form power for both self-selected cadence (power and running economy *r *= 0.2 to 0.8; form power and running economy *r *= 0.1 to 0.8) and lowered cadence (power and running economy *r *= 0.2 to 0.8; form power and running economy *r *= 0.2 to 0.8) conditions. Since the lower and upper confidence limits of the correlations were positive, an inference can be made that there is a clear, substantial relationship between the Stryd Summit’s power metrics and running economy [34], yet the strength of this relationship is unclear. Indeed, confidence intervals for power and running economy at both cadence conditions overlap three or four of Cohen’s [32] scales for interpretation of the magnitude of the positive correlations. 

Aubry, Power, and Burr [27] found a positive, yet weak relationship between power and VO_2_ (*r *= 0.29) pooling data from recreational and elite runners using the Stryd Pioneer chest-mounted running power meter. The authors measured a significant difference in metabolic cost while running on different surfaces, yet this did not elicit a difference in estimated power. The current study was able to measure a significant difference in metabolic cost (paired *t*-test statistic) by altering cadence and a concomitant significant increase in estimated power. 

This study aimed to elicit a significant change in participant running economy and power by attempting to impose a 10% decrease in cadence. The actual decrease in cadence was only 3.9%, yet, one-way paired t-tests reported the lowered cadence (172.5 ± 9.5 strides·min^−1^) was significantly lower (*p *< 0.001) than self-selected cadence (179.6 ± 8.4 strides·min^−1^). Since the relationship between cadence and running economy has been well established [22,23,25], the relatively small change in cadence was less of a concern as running economy was significantly greater with lowered cadence. The significantly lower cadence resulted in significantly greater running economy (self-selected cadence = 201.6 ± 12.8; lowered cadence = 204.5 ± 10.7 mL·kg^−1^·min^−1^), power (self-selected cadence = 4.4 ± 0.5; lowered cadence = 4.4 ± 0.5 W·kg^−1^), and form power (self-selected cadence = 1.1 ± 0.1; lowered cadence = 1.1 ± 0.1 W·kg^−1^). For the purposes of determining the efficacy of the Footpod, the ~4% decrease in cadence was enough to elicit a significant increase in running economy (~1%) and a significant increase (~5%) in form power. 

Although the relationship of power and running economy for both cadence conditions were positive correlations (r values for both cadence conditions were ~0.6), these correlations should be viewed with caution as the corresponding r^2^ value indicates that only ~31% of the variability in running economy can be explained by power. It is also acknowledged that the sample size (*n* = 17) was smaller than ideal for correlation analysis. Although statistically significant correlations were determined for power and running economy, it is not possible to evaluate the strength of the relationship as 90% confidence intervals were large (Table 4 and Table 5). Also, the sample of competitive runners may have already optimized cadence and running biomechanics, thus narrowing the range of variability for running economy correlation. This may be the case in the lower cadence condition as well since SD values were similar for both cadence conditions. While the small sample size and large confidence intervals limit the ability to determine the magnitude of the correlation between power, form power, and running economy at the different running cadences, the viability of the Footpod to provide an indication of changes in running economy outside of a laboratory setting is encouraging. 

While the change in RPE did not reach significance at a *p* < 0.05 level, (*p* = 0.08), it is of interest that runners perceived the lowered cadence condition as a greater effort. The change in ventilation between cadence conditions was also nonsignificant (*p* = 0.77), but the mean difference of 2.2 L·min^−1^ greater ventilation with lower cadence suggest a higher physiological demand. Taking this all into account, lowering cadence from a self-selected value elicited poorer running economy, along with higher power and form power. 

Cadence had negative correlations with form power and negative correlations with both measures of running economy. Previous studies have found a negative relationship between cadence and running economy [23,24,26], although form power may be more sensitive to changes in cadence. In the current study, the percent change in form power from self-selected cadence to lowered cadence was 5.3%, while the percent change in running economy was 1.1%. Form power’s apparent greater sensitivity to cadence changes may be due to increased vertical work necessary for lower cadence running since a lower cadence requires a longer stride length to maintain the same running velocity. Greater vertical displacement of the runner’s center of gravity must occur to produce longer time in the air, so larger vertical ground reaction forces are produced during the contact phase. The larger vertical ground reaction forces produce more negative work during the first half of the contact phase to slow down the runner’s downward velocity at contact and produce more positive work during the second half of the contact phase to speed up the runner’s upward velocity until take off.

Should running power have a large positive correlation with running economy, it may be desirable for runners to monitor power values for improvement both acutely and longitudinally. Previous studies have used various strength-oriented interventions for improving running economy [35], and similar interventions are necessary to test for improvements in power values. Interventions that have improved running economy in previous studies include heavy weight training (squats) [36], explosive strength sessions (sprinting and jumping) [37], and plyometric training [38].

## 5. Conclusions

The present study suggests there is a positive relationship between power and form power, and running economy expressed in terms of oxygen cost and caloric unit cost. It may be that form power is more sensitive to changes in cadence and other biomechanical factors than running economy or caloric unit cost. This is logical since form power is based on mechanical measures from the inertial measurement unit of the Footpod. While target-lowered cadence values were not met, they were statistically less than self-selected cadence values. As expected, the lower cadence resulted in significantly poorer running economy and significantly greater power output at a given treadmill speed. The positive correlation between power and running economy suggests the Footpod may reflect changes in running economy from alterations in cadence yet is not clear if the Footpod is sufficiently accurate to account for differences in the running economy of competitive runners.

## Figures and Tables

**Table 1:** Participant characteristics separated by gender.

	Total Sample (*n* = 17)	Males (*n* = 9)	Females (*n* = 8)
Age (years)	20.6 ± 2.3	19.8 ± 1.9	21.5 ± 2.5
Mass (kg)	62.4 ± 6.9	66.4 ± 6.9	57.9 ± 3.3
Height (cm)	175.0 ± 8.2	180.6 ± 6.4	168.6 ± 4.5
Experience level (years)	7.9 ± 3.2	6.1 ± 2.7	10.0 ± 2.4
Estimated VO_2max _(ml·kg^−1^·min^−1^)	56.6 ± 8.2	63.1 ± 4.6	49.3 ± 3.7
Estimated 5 km time (mm:ss)	18:12 ± 2:19	16:23 ± 1:05	20:17 ± 1:19

Note. Data reported as mean ± standard deviation (SD). Estimated 5 km time corresponds to estimated VO_2max_ values [28].

**Table 2:** Cadence, VO_2_, respiratory exchange ratio, rating of perceived exercitation (RPE), and ventilation by cadence condition.

Condition	Cadence (Strides·min^−1^)	VO_2_ (mL·kg^−1^·min^−1^)	Respiratory Exchange Ratio	RPE	Ventilation (L·min^−1^)
Self-selected cadence	179.6 ± 8.4	52.8 ± 8.7	1.0 ± 0.1	11.7 ± 1.7	93.8 ± 19.3
Lowered cadence	172.5 ± 9.5 *	53.4 ± 8.5 *	1.0 ± 0.1 *	12.1 ± 1.7	96.0 ± 18.4
Percent change	3.9	1.2	2.0	3.9	2.3

Note. Data reported as mean ± SD. * Paired t-test is significant at the *p *< 0.05 level (one-tailed).

**Table 3:** Running economy and power by cadence condition.

Condition	Running Economy (ml·kg^−1^·km^−1^)	Caloric Unit Cost (kcal·kg^−1^·km^−1^)	Stryd Power (W·kg^−1^)	Form Power (W·kg^−1^)
Self-selected cadence	201.6 ± 12.8	1.0 ± 0.1	4.4 ± 0.5	1.1 ± 0.1
Lowered cadence	204.5 ± 10.7 *	1.1 ± 0.1 *	4.4 ± 0.5 *	1.1 ± 0.1 *
Percent change	1.4	1.0	1.1	5.3

Note. Data reported as mean ± SD. * Paired t-test is significant at the *p *< 0.05 level (one-tailed).

**Table 4:** Bivariate correlation matrix of cadence, running economy, caloric unit cost, power, and form power (self-selected cadence condition).

	Cadence	Running Economy	Caloric Unit Cost	Stryd Power	Form Power
Cadence	-				
Running economy	−0.4 (−0.7 to 0.0)	-			
Caloric unit cost	−0.4 * (−0.7 to 0.0)	1.0 ** (0.98 to 1.0)	-		
Stryd power	−0.4 (−0.7 to 0.0)	0.6 ** (0.2 to 0.8)	0.6 ** (0.2 to 0.8)	-	
Form power	−0.8 ** (−0.9 to −0.6)	0.5 * (0.1 to 0.8)	0.5 * (0.1 to 0.8)	0.8 ** (0.5 to 0.9)	-

* Correlation is significant at the *p *< 0.05 level (one-tailed). ** Correlation is significant at the *p *< 0.001 level (one-tailed). 90% confidence intervals reported in parentheses.

**Table 5:** Bivariate correlation matrix of cadence, running economy, caloric unit cost, power, and form power (lowered cadence condition).

	Cadence	Running Economy	Caloric Unit Cost	Stryd Power	Form Power
Cadence	-				
Running economy	−0.5 * (−0.8 to −0.1)	-			
Caloric unit cost	−0.5 * (−0.8 to −0.2)	1.0 ** (0.9 to 1.0)	-		
Stryd power	−0.4 (−0.7 to 0.0)	0.6 * (0.2 to 0.8)	0.5 * (0.2 to 0.8)	-	
Form power	−0.9 ** (−1.0 to −0.7)	0.5 * (0.2 to 0.8)	0.6 ** (0.2 to 0.8)	0.7 ** (0.4 to 0.9)	-

* Correlation is significant at the *p *< 0.05 level (one-tailed). ** Correlation is significant at the *p *< 0.001 level (one-tailed). 90% confidence intervals reported in parentheses.

## References

[1] Woodman J.A., Crouter S.E., Bassett D.R., Fitzhugh E.C., Boyer W.R. (2017). Accuracy of consumer monitors for estimating energy expenditure and activity type. Med. Sci. Sports Exerc..

[2] Meyer J., Hein A. (2013). Live long and prosper: Potentials of low-cost consumer devices for the prevention of cardiovascular diseases. J. Med. Internet Res..

[3] Li R.T., Kling S.R., Salata M.J., Cupp S.A., Sheehan J., Voos J.E. (2015). Wearable performance devices in sports medicine. Sports Health.

[4] Coyle E.F., Feltner M.E., Kautz S.A., Hamilton M.T., Montain S.J., Baylor A.M., Abraham L.D., Petrek G.W. (1991). Physiological and biomechanical factors associated with elite endurance cycling performance. Med. Sci. Sports Exerc..

[5] Sunde A., Støren Ø., Bjerkaas M., Larsen M., Hoff J., Helgerud J. (2010). Maximal strength training improves cycling economy in competitive cyclists. J. Strength Cond. Res..

[6] Moseley L., Jeukendrup A.E. (2001). The reliability of cycling efficiency. Med. Sci. Sports Exerc..

[7] Ettema G., Lorås H.W. (2009). Efficiency in cycling: A review. Eur. J. Appl. Physiol..

[8] Cavagna G., Komarek L., Mazzoleni S. (1971). The mechanics of sprint running. J. Physiol..

[9] Fukunaga T., Matsuo A., Yuasa K., Fujimatsu H., Asahina K. (1980). Effect of running velocity on external mechanical power output. Ergonomics.

[10] Inbar O., Kaiser P., Tesch P. (1981). Relationships between leg muscle fiber type distribution and leg exercise performance. Int. J. Sports Med..

[11] Williams K.R., Cavanagh P.R. (1983). A model for the calculation of mechanical power during distance running. J. Biomech..

[12] Schepens B., Willems P.A., Cavagna G.A., Heglund N.C. (2001). Mechanical power and efficiency in running children. Eur. J. Physiol..

[13] Daniels J. (1985). A physiologist’s view of running economy. Med. Sci. Sports Exerc..

[14] Fletcher J.R., Esau S.P., MacIntosh B.R. (2009). Economy of running: Beyond the measurement of oxygen uptake. J. Appl. Physiol..

[15] Skovgaard C., Christiansen D., Christensen P.M., Almquist N.W., Thomassen M., Bangsbo J. (2018). Effect of speed endurance training and reduced training volume on running economy and single muscle fiber adaptations in trained runners. Physiol. Rep..

[16] Shaw A.J., Ingham S.A., Atkinson G., Folland J.P. (2015). The correlation between running economy and maximal oxygen uptake: Cross-sectional and longitudinal relationships in highly trained distance runners. PLoS ONE.

[17] Saunders P.U., Pyne D.B., Telford R.D., Hawley J.A. (2004). Factors affecting running economy in trained distance runners. Sports Med..

[18] Hunter G.R., McCarthy J.P., Carter S.J., Bamman M.M., Gaddy E.S., Fisher G., Katsoulis K., Plaisance E.P., Newcomer B.R. (2015). Muscle fiber type, Achilles tendon length, potentiation, and running economy. J. Strength Cond. Res..

[19] Williams K.R., Cavanagh P.R. (1987). Relationship between distance running mechanics, running economy, and performance. J. Appl. Physiol..

[20] Hoogkamer W., Kipp S., Spiering B., Kram R. (2016). Altered running economy directly translates to altered distance-running performance. Med. Sci. Sports Exerc..

[21] Hoogkamer W., Kipp S., Frank J.H., Farina E.M., Luo G., Kram R. (2017). A comparison of the energetic cost of running in marathon racing shoes. Sports Med..

[22] Cavanagh P.R., Pollock M.L., Landa J. (1977). A biomechanical comparison of elite and good distance runners. Ann. N. Y. Acad. Sci..

[23] Tartaruga M.P., Brisswalter J., Peyré-Tartaruga L.A., Ávila A.O., Alberton C.L., Coertjens M., Cadore E.L., Tiggemann C.L., Silva E.M., Kruel L.F. (2012). The relationship between running economy and biomechanical variables in distance runners. Res. Q. Exerc. Sport.

[24] Halvorsen K., Eriksson M., Gullstrand L. (2012). Acute effects of reducing vertical displacement and step frequency on running economy. J. Strength Cond. Res..

[25] De Ruiter C.J., Verdijk P.W.L., Werker W., Zuidema M.J., de Haan A. (2014). Stride frequency in relation to oxygen consumption in experienced and novice runners. Eur. J. Sport Sci..

[26] Lieberman D.E., Warrener A.G., Wang J., Castillo E.R. (2015). Effects of stride frequency and foot position at landing on braking force, hip torque, impact peak force and the metabolic cost of running in humans. J. Exp. Biol..

[27] Aubry R., Power G.A., Burr J.F. (2018). An assessment of running power as a training metric for elite and recreational runners. J. Strength Cond. Res..

[28] Daniels J. (2014). Daniels Running Formula.

[29] Daniels J., Gilbert J. (1979). Oxygen Power: Performance Tables for Distance Runners.

[30] Daniels J., Daniels N. (1992). Running economy of elite male and elite female runners. Med. Sci. Sports Exerc..

[31] Morgan D.W., Bransford D.R., Costill D.L., Daniels J.T., Howley E.T., Krahenbuhl G.S. (1995). Variation in the aerobic demand of running among trained and untrained subjects. Med. Sci. Sports Exerc..

[32] Cohen J. (1988). Statistical Power Analysis for the Behavioral Sciences.

[33] Fisher R.A. (1921). On the probable error of a coefficient of correlation deduced from a small sample. Metron.

[34] Batterham A.M., Hopkins W.G. (2006). Making meaningful inferences about magnitudes. Int. J. Sports Physiol. Perform..

[35] Denadai B.S., Aguiar R.A., Lima L.C., Greco C.C., Caputo F. (2016). Explosive training and heavy weight training are effective for improving running economy in endurance athletes: A systematic review and meta-analysis. Sports Med..

[36] Storen O., Helgerud J., Stoa E.M., Hoff J. (2008). Maximal strength training improves running economy in distance runners. Med. Sci. Sports Exerc..

[37] Paavolainen L., Häkkinen K., Hämäläinen I., Nummela A.R.I., Rusko H. (1999). Explosive-strength training improves 5-km running time by improving running economy and muscle power. J. Appl. Physiol..

[38] Berryman N., Maurel D., Bosquet L. (2010). Effect of plyometric vs. dynamic weight training on the energy cost of running. J. Strength Cond. Res..

